# Structural evidence for the covalent modification of FabH by 4,5-dichloro-1,2-dithiol-3-one (HR45)†
†Electronic supplementary information (ESI) available. See DOI: 10.1039/c7ob01396e


**DOI:** 10.1039/c7ob01396e

**Published:** 2017-07-17

**Authors:** Alexander G. Ekström, Van Kelly, Jon Marles-Wright, Scott L. Cockroft, Dominic J. Campopiano

**Affiliations:** a EaStCHEM School of Chemistry , University of Edinburgh , Joseph Black Building , David Brewster Road , Edinburgh , EH9 3FJ , UK . Email: Dominic.Campopiano@ed.ac.uk; b School of Biological Science , Newcastle University , Devonshire Building , Newcastle upon Tyne , NE1 7RU , UK

## Abstract

We use mass spectrometry analysis and molecular modelling to show the established antimicrobial inhibitor 4,5-dichloro-1,2-dithiol-3-one (HR45) acts by forming a covalent adduct with the target β-ketoacyl-ACP synthase III (FabH). The 5-chloro substituent directs attack of the essential active site thiol (C112) *via* a Michael-type addition elimination reaction mechanism.

## Abstract 2

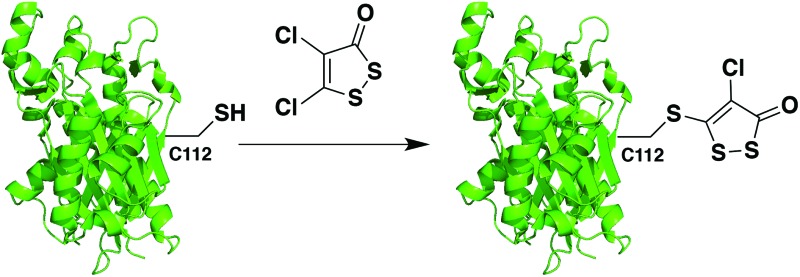
Mass spectrometry and modelling shows the antimicrobial inhibitor 4,5-dichloro-1,2-dithiol-3-one (HR45) acts by forming a covalent adduct with the target β-ketoacyl-ACP synthase III (FabH). The 5-chloro substituent directs attack of the essential active site thiol (C112) *via* a Michael type addition elimination reaction mechanism.

## 

Fatty acid biosynthesis is essential for cell viability and growth, deriving fatty acids from the fundamental building block acetyl-CoA. In eukaryotes, this process is facilitated by type I fatty acid synthase (FAS I); a large, multi-domain enzyme containing all required functionalities.^1^ In contrast, most bacteria utilise a complex of proteins termed type II fatty acid synthase (FAS II), whereby each functionality is carried out by a discreet enzyme, between which the growing acyl chain is transported by the acyl carrier protein (ACP).^1^ Substantial differences in the architecture and chemistry carried out by FAS I and II systems has led to significant interest in the bacterial pathway as a target for new antibacterial compounds.

β-Ketoacyl-ACP synthase III (FabH) is the first condensing enzyme in the FAS II pathway,^2^ and has attracted significant attention as a target for novel antibiotic design.^3^–^7^ This ubiquitous, highly conserved enzyme catalyses the cysteine-mediated, Claisen-like condensation between malonyl-ACP and short chain acyl-CoAs. Despite the suggestion that Gram-positive bacteria can absorb and utilise exogenous fatty acids,^8^,^9^ FabH is widely believed to be essential for cell viability.^10^ A few natural product inhibitors of FAS II exist in the literature,^11^,^12^ but despite research efforts there are no FabH-specific inhibitors approved for clinical use.

Of the natural products, the thiolactone antibiotic thiolactomycin (TLM, Fig. 1) has selectivity for FAS II condensing enzymes by mimicking malonyl-ACP binding.^11^,^13^ He *et al.* used TLM as a starting point to develop potent FabH inhibitors by searching the National Cancer Institute (NCI) database for structurally similar molecules.^3^,^14^,^15^ The most potent hit was 4,5-dichloro-1,2-dithiol-3-one (also referred to in the literature as HR45 and DDCP, Fig. 1). Subsequent structure–activity relationship studies showed that the chlorine in the 5-position was found to be essential for irreversible inhibition of FabH isoforms from both *Staphylococcus aureus* (*sa*FabH) and *Escherichia coli* (*ec*FabH) with reported IC_50_ values of 156 nM and 2.0 μM, respectively.^15^ The reported mode of inhibition was inconclusive, and despite postulating covalent modification *via* a Michael-type mechanism they were unable to obtain data to support this hypothesis. This commercially available 1,2-dithiol-3-one (CAS 1192-52-5) has since been frequently reported as a positive control for FabH inhibition,^16^–^21^ and also has FDA approval as a slimicide additive in the paper industry for food packaging (FDA docket number 99F-1423).

**Fig. 1:**
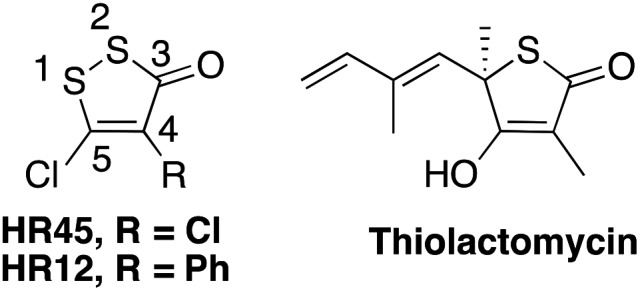
HR45 and HR12, hits against *sa*FabH and *ec*FabH from an NCI database screen by He *et al.* based on thiolactomycin.^15^ HR12 identified as a hit against *ec*MurA in a separate study.^22^

A less efficacious hit from the same study was HR12 (also known as RWJ-3981, Fig. 1), an analogue of HR45 with a phenyl group replacing the chlorine in the 4-position, with reported IC_50_ values of 0.98 μM and 5.7 μM for *sa*FabH and *ec*FabH respectively.^3^,^15^ Interestingly, in a separate study HR12 was identified as a hit against *E. coli* uridine diphosphate-*N*-acetyl glucosamine enolpyruvyl transferase (*ec*MurA), a cysteine-dependent enzyme involved in the first committed step of peptidoglycan cell wall biosynthesis.^22^

The mode of inhibition was speculated to be covalent modification of the catalytic *ec*MurA C115 residue. Due to the lack of homology between *sa*FabH and *ec*MurA, it is unlikely that the HR45 mechanism is restricted to these two enzymes.^15^ The authors were unable to obtain supporting mass spectrometry (MS) data and suggested that the modification did not survive the MS conditions. A key point to note was that the inhibition could be reversed through reduction of the disulfide by dithiothreitol (DTT).

The structure of HR45 suggests that the Michael-type acceptor nature of the cyclic α,β-unsaturated ketone could define it as a pan assay interference compound (PAIN)^23^ with non-specific activity towards nucleophilic amino acid residues. Whilst PAINs are often removed from traditional small molecule screens, it has been suggested that this should not be the case when screening for antimicrobial activity.^24^ To begin our study of the 1,2-dithiol-3-ones we conducted a simple model reaction between HR45 and *N*-acetylcysteine (Fig. S1 and 2†). The reaction was followed by ^1^H NMR, showing an immediate shift in the pro-chiral CH_2_ group (Fig. S2†). Subsequent ^13^C and 2D HMBC NMR experiments confirmed that the addition–elimination had taken place at the 5-position (Fig. S2†). This proposed mechanism is supported by Spartan modelling of HR45, which indicated C-5 as the most electrophilic centre (Fig. S3†). Encouraged by this data we set out to capture FabH-bound adducts of HR45 by MS.

We elected to work with *sa*FabH, an isoform with only one catalytically essential cysteine residue, against which HR45 had shown the highest activity.^15^ Recombinant *sa*FabH was isolated from *E. coli* and found to be gluconated, as is often observed on N-terminal hexahistidine affinity tags.^25^ Removal of the tag was essential to produce uncomplicated LC-MS spectra of the intact protein (Fig. S8†). This resulted in well-resolved MS data with a single peak for each charge state and a deconvoluted mass of 34 063.7 Da that matched well with predicted values (Fig. 2a and Fig. S8†). Covalent modification of *sa*FabH by HR45 was rapid and quantitative in the absence of reducing agent, accompanied with a shift of the deconvoluted mass of +150 Da (Fig. 2b). This mass change is consistent with the addition of HR45 with the loss of HCl. This data supports our proposed mechanism, involving attack of the nucleophilic cysteine residue followed by elimination of the *ipso*-chlorine (Fig. S2†). The HR45 modification was quantitatively removed by incubation with excess DTT (2 mM) for 15 min, resulting in the native protein mass. We also treated the native *sa*FabH with the irreversible, cysteine-specific label *N*-ethylmaleimide (NEM), which resulted in a mass shift of +125 Da, signifying quantitative labelling of a single cysteine residue (Fig. 2c). The NEM-modified sample was then incubated with HR45 for 3 h; resulting in no further mass change (Fig. 2d). This indicates that alkylation of C112 prevents the reaction of *sa*FabH with HR45 (Fig. 2d).

**Fig. 2:**
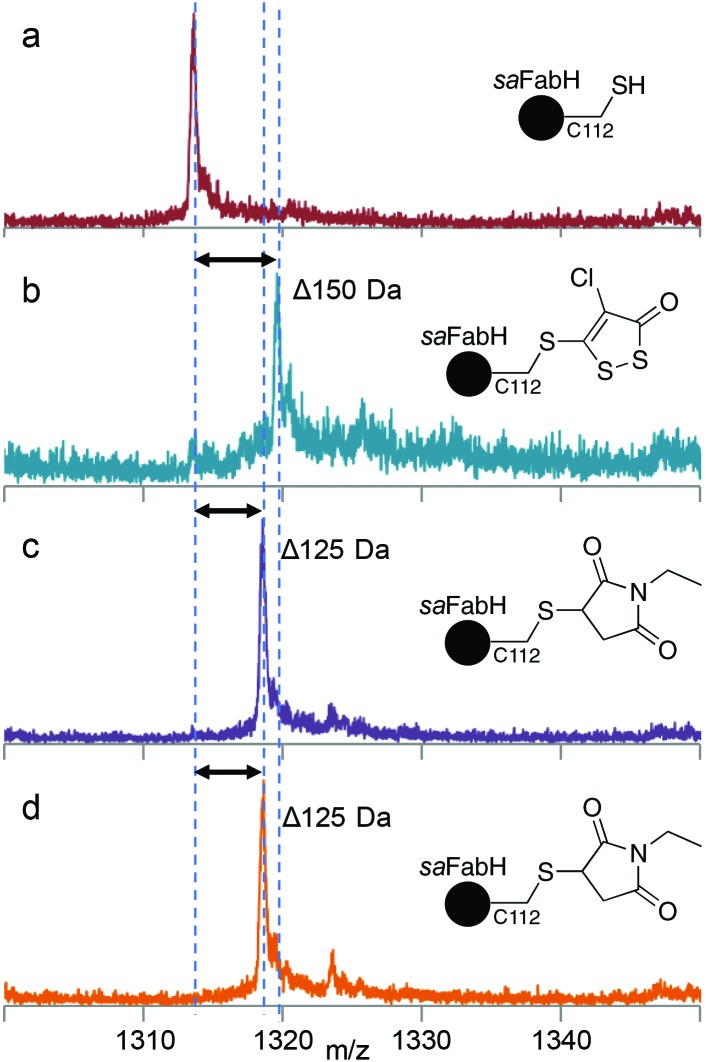
The LC-MS spectra (26+ charge state) of *sa*FabH (a) unmodified control, (b) protein incubated with HR45 for 30 min at RT resulting in complete modification (+150 Da), (c) protein incubated with *N*-ethylmaleimide (NEM) resulting in complete alkylation of the single cysteine residue (+125 Da), and (d) NEM-alkylated protein incubated with HR45 for 3 hours at RT resulting in no mass change.

To further confirm modification of C112, HR45-modified *sa*FabH was digested with trypsin to generate peptide fragments. As well as the absence of the ion corresponding to the C112-containing tryptic peptide (103-VASMDQLAACSGFMYSMITAK-123, predicted monoisotopic mass 2224.0036 Da, observed 1113.0042 Da [M + 2H]^2+^) in the control digest (Fig. 3a), we also failed to detect a peptide corresponding to the expected mass of the C112-HR45 adduct. This observation was consistent with previous studies that attempted to detect HR12 adducts.^22^ To discover the fate of the C112-containing peptide, it was purified from a large-scale trypsin digest of *sa*FabH with activated thiol sepharose. After elution with DTT, the peptide was desalted and resuspended in either ammonium bicarbonate (pH 8.0) or ammonium acetate (pH 7.0) buffer and incubated in the presence or absence of HR45 to investigate the stability of the peptide and the HR45 adduct. In ammonium acetate, both the unmodified and HR45-modified forms were observed at the predicted masses (Fig. 3a and c), consistent with observations on the intact protein (Fig. 2b). In contrast, in ammonium bicarbonate (Fig. 3b), the unmodified peptide was found to exist predominantly as a [M + 4H]^4+^ disulfide dimer. Perplexingly, upon incubation of the HR45-modified form in ammonium bicarbonate (Fig. 3d) a new signal appeared corresponding to a loss of 34 Da from the unmodified peptide (predicted 2189.9837 Da, observed 1095.9991 Da [M + 2H]^2+^). Using CID tandem MS, we obtained a fragmentation spectrum which we interpreted as the conversion of C112 to dehydroalanine (Dha) (Fig. S7†). A small amount of this Dha-containing peptide also formed from the disulfide dimer at pH 8.0 (Fig. 3b).

**Fig. 3:**
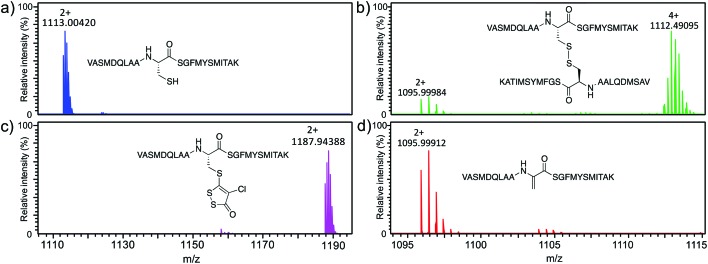
Purified tryptic peptide (103-VASMDQLAACSGFMYSMITAK-123) in (a) ammonium acetate (100 mM, pH 7.0) for 16 h at 37 °C and (b) ammonium bicarbonate (100 mM, pH 8.0) for 16 h at 37 °C. The purified peptide modified with HR45 in (c) ammonium acetate (100 mM, pH 7.0) for 16 h at 37 °C, and (d) in ammonium bicarbonate (100 mM, pH 8.0) for 16 h at 37 °C. All masses were observed within 15 ppm of expected.

This C112 to Dha conversion under typical tryptic digest conditions may be the reason for previous failures to observe the covalent modification of *sa*FabH and *ec*MurA by HR45 and similar inhibitors. The alkaline hydrolysis pathway of 1,2-dithiol-3-ones is a highly complex one.^26^ Although we do not yet have sufficient data to speculate on the mechanism of the C112-HR45 to Dha conversion, it is likely that one of the hydrolysis products facilitates the transformation, possibly by a similar mechanism to other Dha formation chemistry.^27^

The instability of the adduct under standard trypsin digest conditions led us to seek an alternative method. We digested HR45-modified *sa*FabH at pH 2.0 with pepsin, and identified two peptic peptides in the unmodified control, AACSGF and AACSGFM^Ox^ (M^OX^ denotes oxidation of the M residue). The extracted ion chromatogram (EIC, Fig. 4) shows the two unmodified peptic fragments that are absent in the HR45-modified sample. In the modified sample, two new signals appear in the EIC with masses corresponding to AAC*SGF and AAC*SGFM^Ox^, where C* denotes a cysteine modification of +150 Da, corresponding to addition of HR45 with the loss of HCl. Clearly a chlorine atom is incorporated in the modified peptides due to the distinctive isotope pattern (Fig. 4). CID tandem MS was used to fragment AAC*SGFM^Ox^, confirming that the modification was indeed on the cysteine residue (Fig. S6†). When similar pH-dependence experiments were conducted on the intact *sa*FabH-HR45 adduct, we observed no evidence of dehydroalanine formation. In the intact protein, C112 is located at the N-terminus of a long α-helix which lowers the p*K*_a_ of C112 from 8.8 to 7.2.^28^,^29^ What impact this environment has on the stability of the HR45 adduct requires further study.

**Fig. 4:**
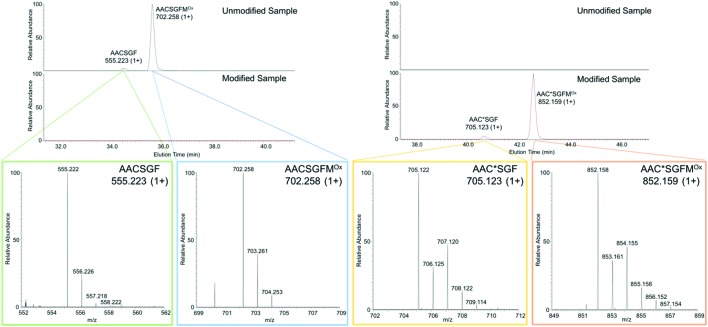
The EICs of C112-containing peptides after pepsin digest. Left: Unmodified *sa*FabH with expanded MS spectra of two separate unmodified peptides. Right: HR45-modified *sa*FabH with expanded MS spectra of two separate modified peptides. Note the appearance of the chlorine isotope pattern in both modified ions.

Despite quantitative modification, we were unable to obtain crystallographic data of HR45-modified *sa*FabH. We modelled the adduct by docking the recent crystal structure of HR45 30 into the active site of *sa*FabH^31^ (PDB: ; 3IL7) guided by our proposed mechanism (Fig. S2 and S4†). Although there is ample space for HR45 in the active site, the catalytically essential residue C112 has little freedom of movement, limiting the likely positions of the ligand and suggesting possible hydrogen bonding between the C-5 oxygen of HR45 and the side chain of the catalytically essential N268.^29^ This model could be a starting point to develop highly specific inhibitors targeting FabH.^32^

## Conclusions

We have shown that the 1,2-dithiol-3-one HR45 covalently modifies *sa*FabH through a Michael-type addition elimination at the catalytic residue C112. We suggest that this is also the mode of inhibition of the molecule on other FabH isoforms, and potentially other systems including the inhibition of *ec*MurA by HR12. We have also shown that the reason that this covalent modification has previously escaped detection is that under normal tryptic digest conditions the alkaline hydrolysis pathway of HR45 facilitates the conversion of the C112 to Dha, the mechanism of which remains to be determined. Describing the precise mode of action of molecules isolated from screening campaigns that failed to make the clinic may have impact on the design and discovery of new antimicrobial leads.^24^ Our detailed mechanistic study of HR45 provides insight for the design of new antimicrobials, and this approach will no doubt be useful for other systems.

## Supplementary Material

Supplementary information
