# Supplementary material for: Structural evidence for the covalent modification of FabH by 4,5-dichloro-1,2-dithiol-3-one (HR45)
Source: Org Biomol Chem. 2017 Jul 17;15(30):6310–3. doi: 10.1039/c7ob01396e (PMC5708339; doi:10.1039/c7ob01396e)
Supplement: Supplementary file 1 [file OB-015-C7OB01396E-s001.pdf]

Electronic Supplementary Material (ESI) for Organic & Biomolecular Chemistry.
This journal is © The Royal Society of Chemistry 2017

Supplementary Information for:

Structural evidence for the covalent modification of FabH by 4,5-dichloro-1,2-dithiol-3-one (HR45)

Alexander G. Ekström[a], Van Kelly[a], Jon Marles-Wright[b], Scott L. Cockroft[a], and Dominic J. Campopiano[a]

a EaStCHEM School of Chemistry, University of Edinburgh, Joseph Black Building, David Brewster Road, Edinburgh, EH9 3FJ, UK.

b School of Biological Science, Newcastle University, Devonshire Building, Newcastle upon Tyne, NE1 7RU, UK.

Contents:

NMR Data	S2-S4
DFT Calculations	S5
Molecular Modelling	S6
MS Data	S7-S10
Microbiology Experimental	S11-S14
References	S15

Primary data files can be found at:

http://dx.doi.org/10.7488/ds/2103

Nuclear magnetic resonance (NMR) spectra were recorded at 298 K on Bruker PRO500 or AVA500 spectrometers running at 500 MHz (^{1}H spectra) or 126 MHz (^{12}C spectra). Chemical shift values (δ) are reported in parts per million (ppm) relative to tetramethylsilane (δ_{TMS} = 0) and are referenced to the residual solvent peak. ^{1}H NMR data are reported in the format: chemical shift, relative intensity, multiplicity (s = singlet, d = doublet, t = triplet, m = multiplet, br = broad), coupling constant (*J* value, Hz), and assignment. ^{13}C NMR data are reported in the format: chemical shift and assignement. ^{1}H/^{13}C HMBC NMR data are reported qualitatively.

<u>Fig. S1: HR45 NMR</u>

HR45

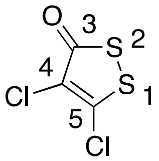

^{13}C NMR (126 MHz, DMSO-*d*$_6$) δ 121.48 (C-4), 153.7 (C-5), 184.6 (C-3)

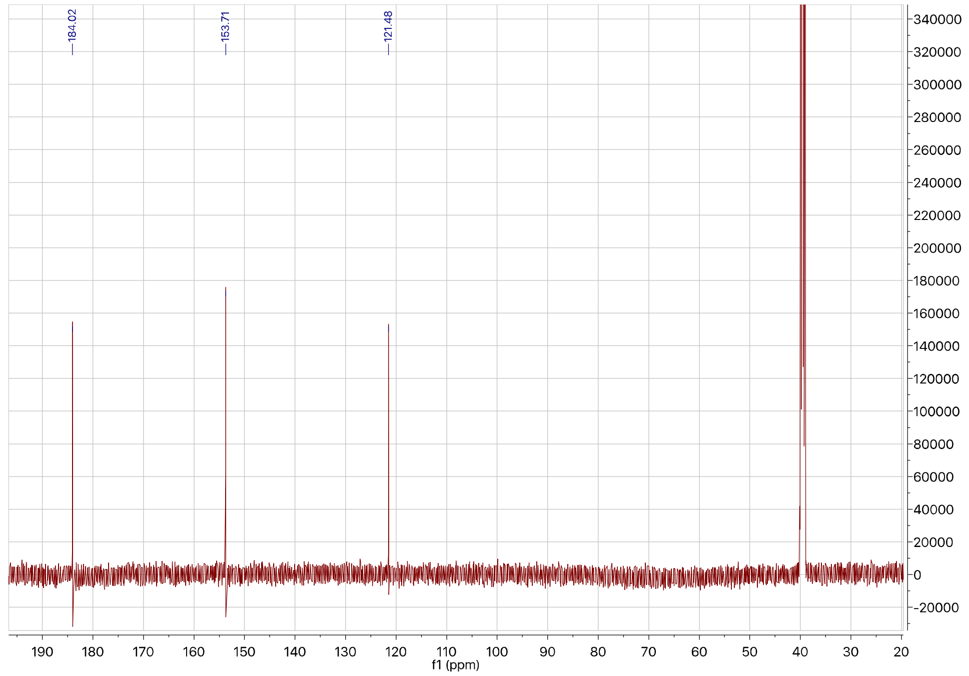

Fig. S2: Model Reaction of HR45 with N-acetylcysteine and NMR spectra

General mechanism:

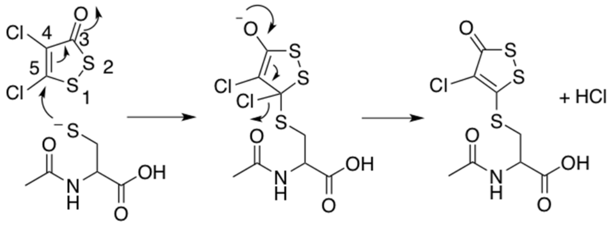

HR45/*N*-acetylcysteine adduct

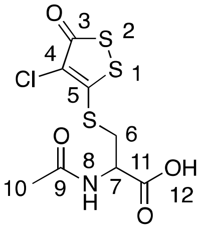

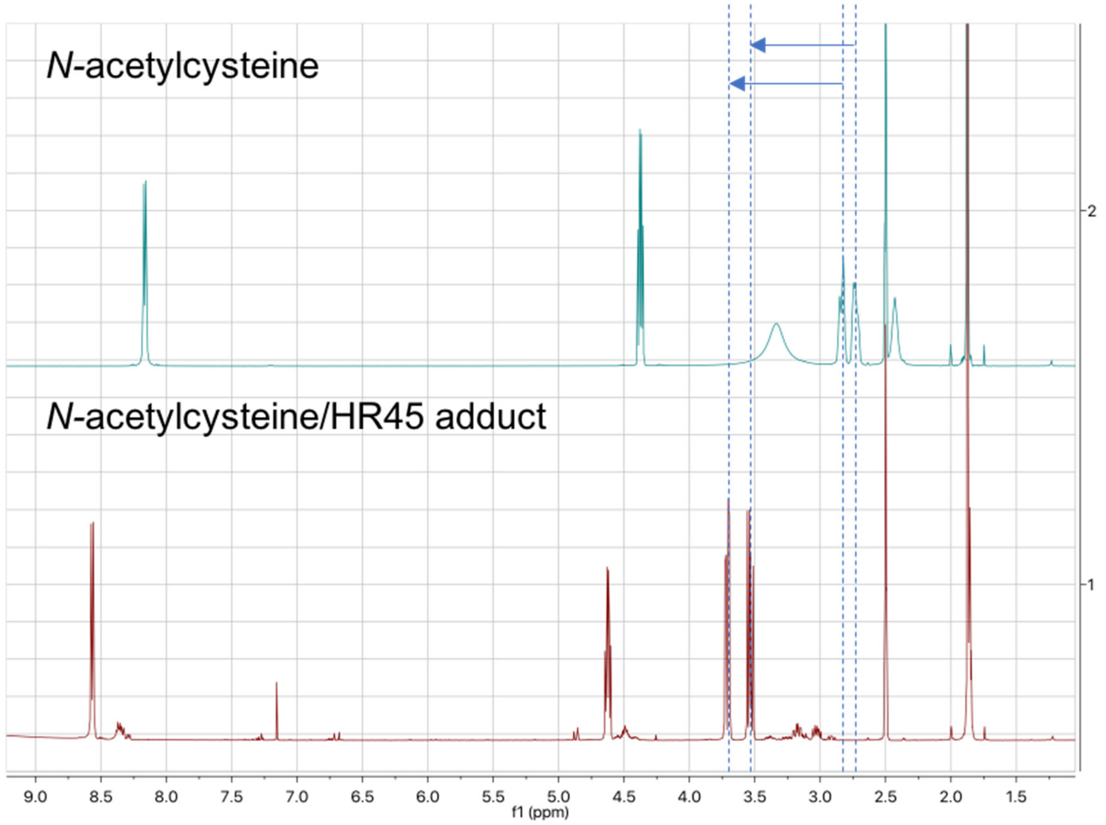

^{1}H NMR (500 MHz, DMSO-d_6) 1.87 (3H, s, CH_3-10), 3.55 (1H, dd, ^2J = 13.5 Hz, ^3J = 8.5 Hz, CH_2-6), 3.70 (1H, dd, ^2J = 13.5 Hz, ^3J = 4.5 Hz, CH_2-6'), 4.59-4.65 (1H, m, CH-7), 8.54 (1H, d, ^3J = 8.1 Hz, NH-8), 9.65 (1H, s, OH-12)

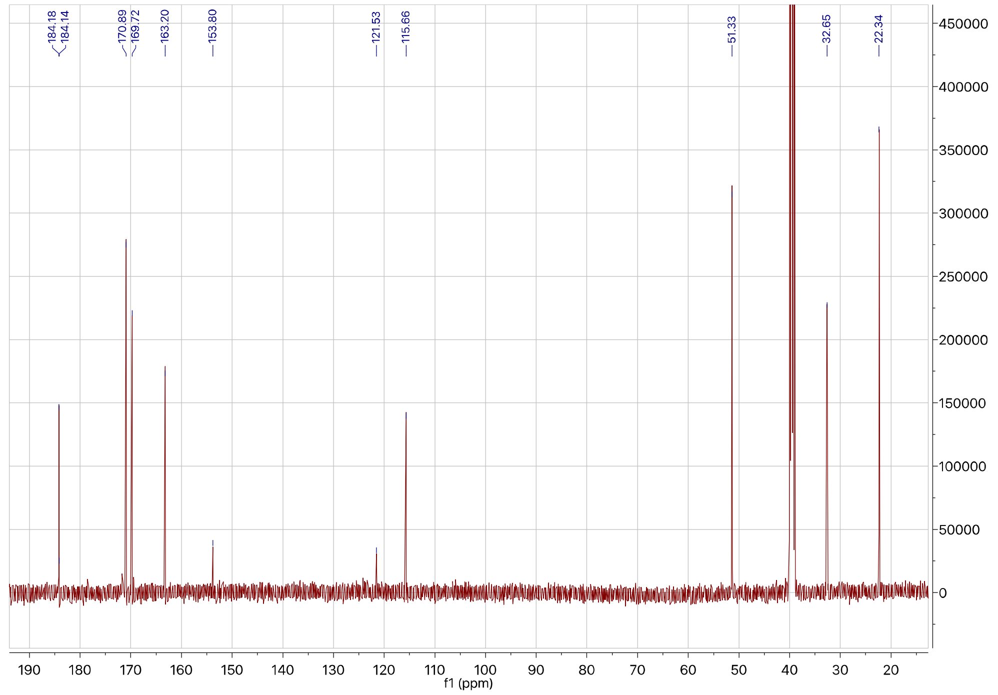

^{13}C NMR (126 MHz, DMSO-d_6) δ 22.3 (CH_3-10), 32.7 (CH_2-6), 51.3 (CH-7), 115.7 (C-4), 163.2 (C-5), 169.7 (C-9), 170.9 (C-11), 184.3 (C-3)

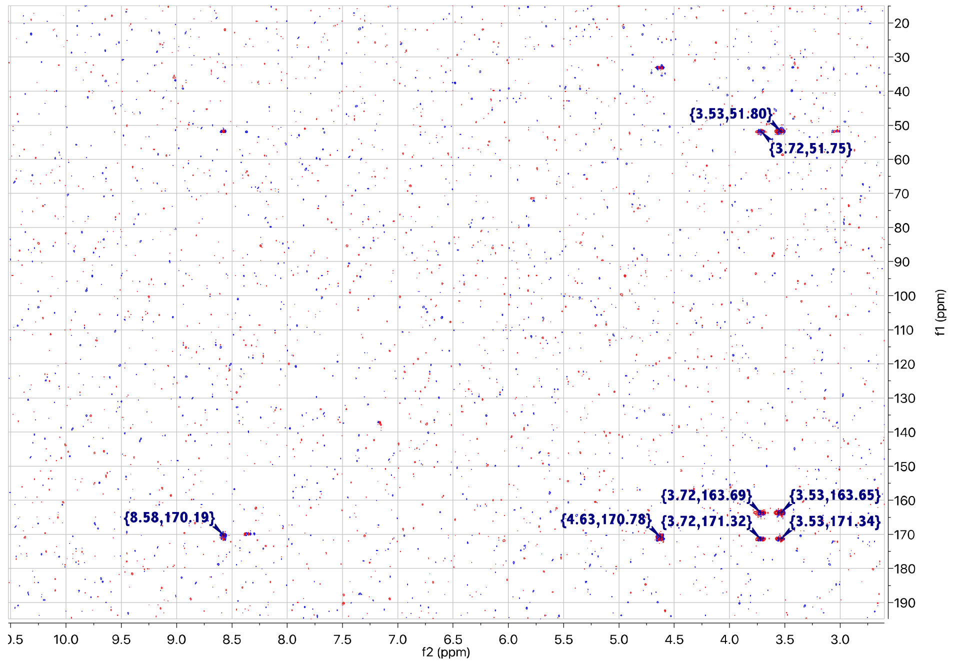

^{1}H^{13}C HMBC

Cross-peaks between C-5 and CH_2-6 (3.53, 163.7) and CH_2-6’ (3.72, 163.7), and absence of cross-peaks between C-4 and CH_2-6 and CH_2-6’ confirm that the addition-elimination reaction has taken place at the C-5 position.

DFT calculations support nucleophilic attack at C5 of HR45/DDCP

The structure of HR45 was minimised using B3LYP/6-311G*. The LUMO and LUMO+1 orbitals were degenerate in energy. The LUMO+1 is shown below and is consistent with a substrate that would undergo Michael addition at C5. According to frontier molecular orbital theory, the shape of this orbital should approximate the localisation of the additional pair of electrons following nucleophilic attack of the S atom of Cys112 on HR45. The largest orbital coefficient (lobe) associated with a single atom is located over C5, consistent with the site that would favour formation of a new σ-bond (i.e. between C5 of HR45 and the S atom of Cys112). Furthermore, this orbital (and therefore, the pair of electrons accepted during the addition reaction) is further delocalised over C4 and C3 (with double-bond character) and onto the carbonyl oxygen, consistent with the enolate intermediate formed during the proposed addition reaction shown in the centre of Figure 2 in the main text.

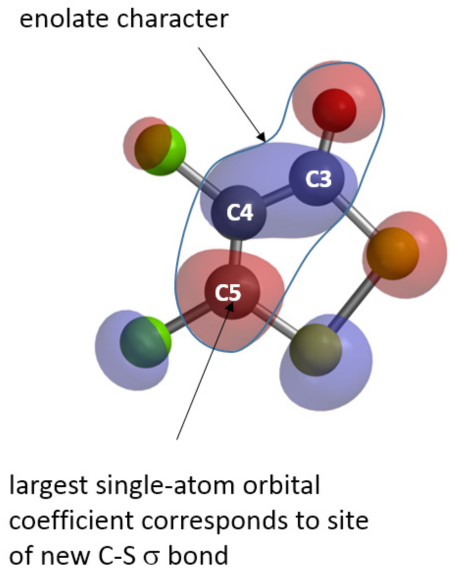

Fig. S3: Localisation of LUMO+1 of HR45 calculated using B3LYP/6-311G* indicating Michael acceptor character.

Model of HR45 small molecule crystal structure docked into saFabH protein crystal structure (PDB:3IL7)

The X-ray structure of HR45 was obtained from the CCDC (CCDC Number: 1517056)[1]. The coordinates for a Cysteine-HR45 C5-SG adduct were produced using JLigand[2]; and the adduct was then manually docked into the crystal structure of saFabH to replace Cys112 (PDBID: 3IL7)[3] and adjusted to minimise steric clashes and to maximize hydrogen bonding potential using coot[4]. The resulting model was then energy minimized using refmac5[5]. Model images were produced using PyMol (Schrödinger LLC).

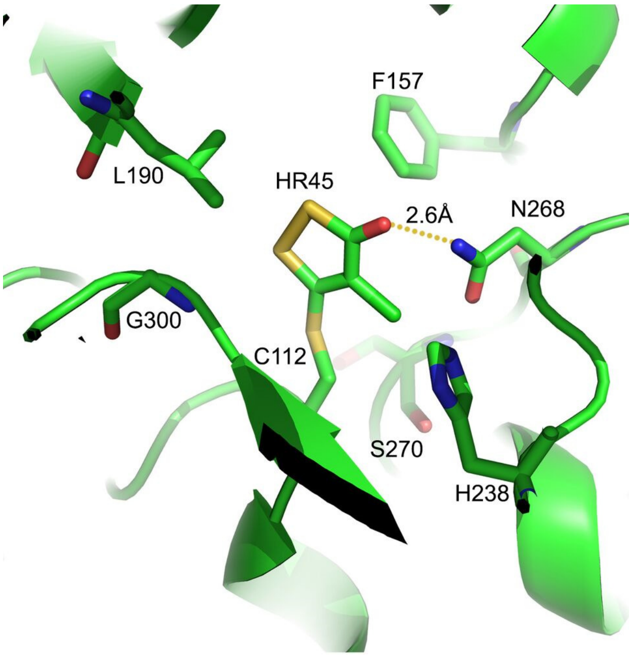

Fig. S4: Model of HR45 small molecule crystal structure docked into saFabH protein crystal structure (PDB:3IL7)

Mass Spectrometry Data and Methods

Purified tryptic peptide samples were analysed by direct infusion electrospray ionisation using a TriVersa nanomate (Advion) coupled to a Bruker 12T SolariX with a 10cm infinity cell. Spectra were acquired in positive polarity over 100-2000m/z with a 2megaword data size. 20 spectra were summed, each with a 50 ms accumulation time. Pepsin digests were analysed by C18 LC-MS/MS over a 50 min gradient from 5% to 45% acetonitrile/0.1% formic acid. MS spectra were acquired on an LTQ Orbitrap in positive polarity over a 333-1800 m/z range using a 371.10124 lockmass. MS scans were acquired at 60k resolution followed by 6 data depended ion trap MSMS spectra, or at 30k resolution followed by 4 7500 resolution Orbitrap MSMS spectra. In additional analyses, candidate modified peptides were fragmented in the Orbitrap, as above, but using a data independent inclusion list. MSMS spectra were converted to mgf format and peptides assigned using the Mascot search engine (www.matrixscience.com).

Peptic Digest Analysis

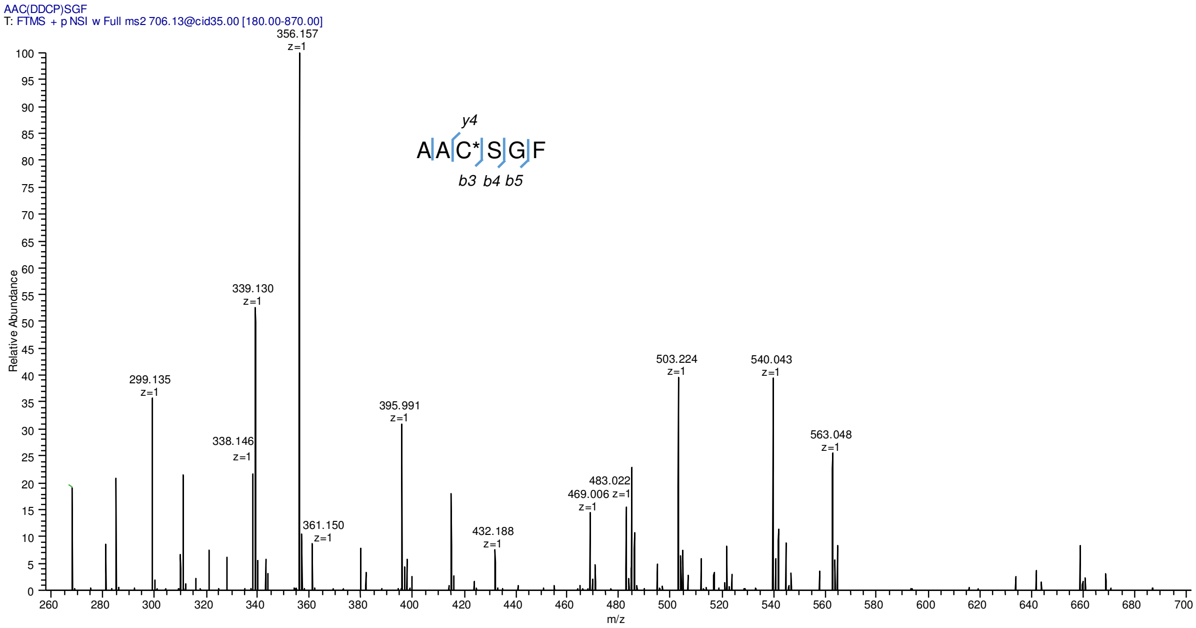

299.1353455	*B4-nHR45*
338.1459045	*B5-nHR45-water*
339.1297302	*B5-nHR45-ammonia*
356.1565552	*B5-nHR45*
361.1502686	*P-nHR45-H2O-Ala-Ala*
395.9905701	*B3*
432.1878052	*P-nHR45-water-Ala*
483.0220337	*B4*
503.2238464	*P-nHR45-water*
540.0432739	*B5*
563.0484619	*Y4*

Fig. S5: MS^2 of peptic peptide 706.13, AAC*SGF and assignment

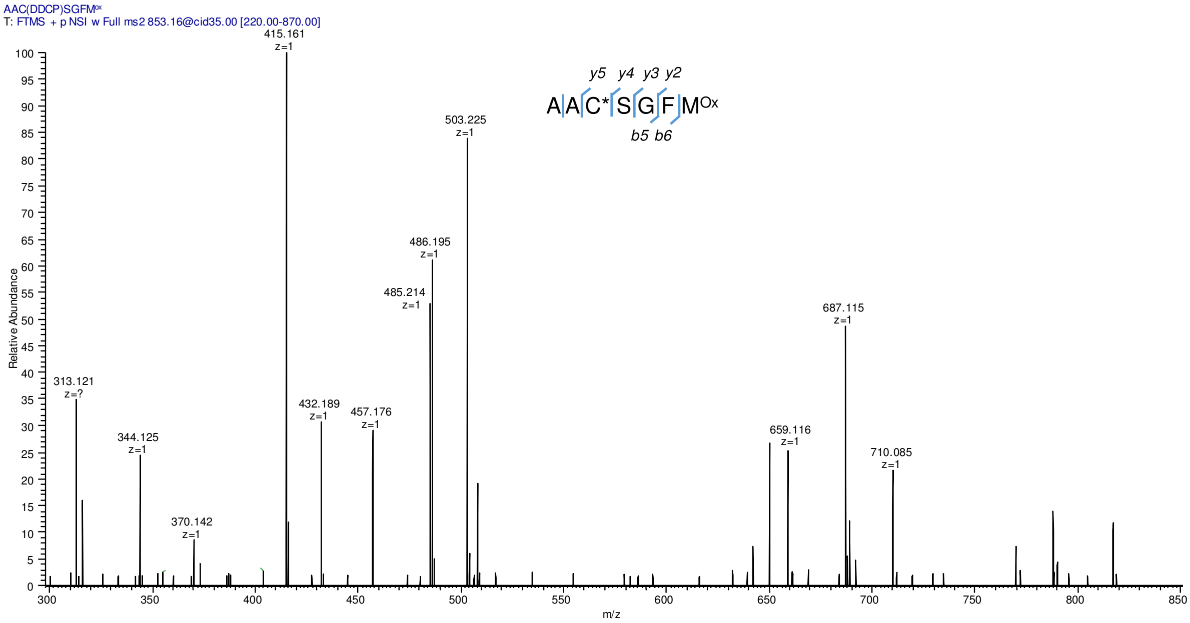

313.1210327	*Y2*
344.1246033	*B5-nHR45-ammonia-Ala*
370.1424561	*Y3*
415.1611023	*B5-nHR45-ammonia*
432.1890259	*B6-nHR45-ammonia-Ala*
457.1759338	*Y4*
485.214325	*B6-nHR45-water*
486.195282	*B6-nHR45-ammonia*
503.2246094	*B6-nHR45*
659.1160889	*B5-CO*
687.1149292	*B6*
710.0846558	*Y5*

Fig. S6: MS^2 of peptic peptide 853.16, AAC*SGFM^{Ox} and assignment

Purified Tryptic Peptide

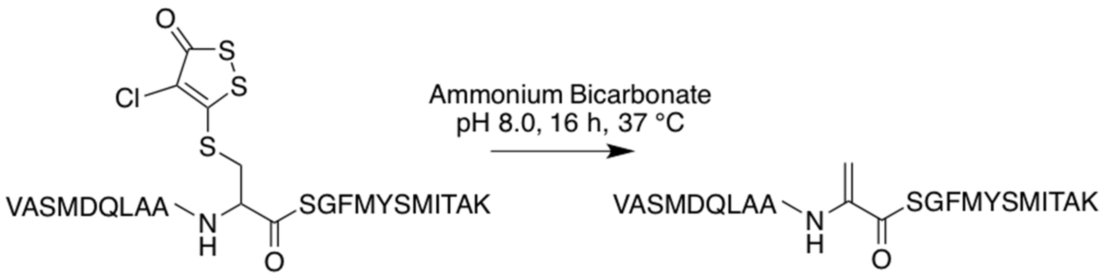

MS^2 of purified tryptic peptide 3+ charge state (731.0126 Da), after incubation with HR45 overnight at 37 °C in 100 mM ammonium bicarbonate (pH 8.0) resulting in C112→Dha conversion. Image from Mascot database search (www.matrixscience.com).

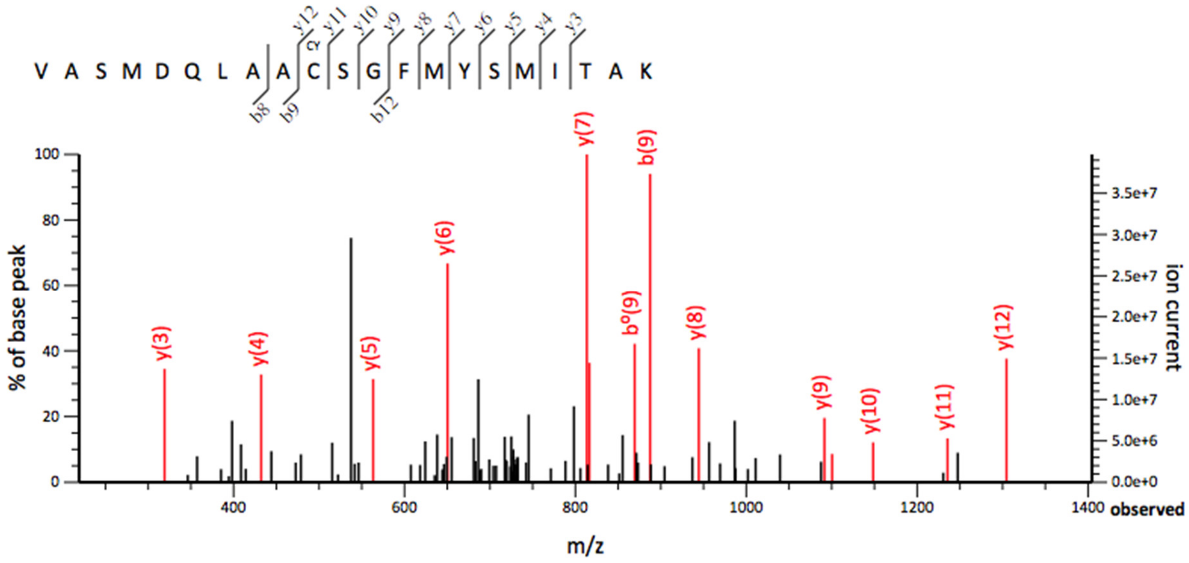

Fig. S7: MS^2 fragmentation of purified tryptic peptide/HR45 adduct after Dha forming conditions

Protein expression and purification, modification and digest protocols

Modification of saFabH with HR45

A stock solution of HR45 (25 mM) in DMSO was always prepared immediately before use. In a typical modification experiment, HR45 (25 mM, DMSO) was added to an aliquot of *sa*FabH (12 µM) such that the final concentration of HR45 was 250 µM with a final concentration of 1% DMSO (v/v). The solution was mixed thoroughly and left for 0.5 h at 37 °C.

Trypsin digest and thiol bead purification

*sa*FabH (2.5 mL, 0.4 mg/mL) in non-reducing buffer (20 mM Tris-HCl pH 7.6, 300 mM NaCl, 10% glycerol) was incubated with trypsin (40 µL, 0.5 mg/mL, Promega) overnight at 37 °C with shaking at 300 rpm. The reaction was then incubated with 4B-activated thiol resin (100 mg dry weight, pre-equilibrated in non-reducing buffer) and slowly rotated for 1 h. The slurry was then added to a 1 mL spin-cartridge fitted with a frit. The slurry was washed with non-reducing buffer (3 x 400 µL) before the bound cysteine-containing peptide was eluted with elution buffer (200 µL, 20 mM DTT, 20 mM Tris-HCl pH 7.6, 300 mM NaCl, 10% glycerol). The eluate was then desalted into MeCN:H_2O (2:1, 0.1% formic acid, 200 µL) using a C18 SPE cartridge and used immediately or lyophilised and stored at -20 °C.

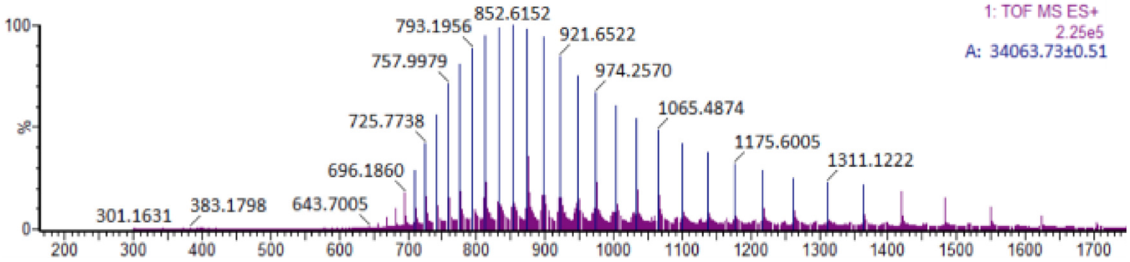

Fig. S8: Full LCMS spectrum of tag-less *sa*FabH

Pepsin digest

To an aliquot of *sa*FabH (2.5 mL, 0.4 mg/mL) in non-reducing buffer (20 mM Tris-HCl pH 7.6, 300 mM NaCl, 10% glycerol) was added HCl (100 µL, final conc. 0.04 N). Lyophilised pepsin (Promega) was resuspended in ddH_2O (1 mg/mL, pH 5.5), and added (25 µL, 1:40 w:w) to the acidified protein aliquot and incubated overnight at 37 °C with shaking at 300 rpm. The reaction was stopped by heating to 95 °C for 10 minutes. The peptic digest was then desalted

into MeCN:H_2O (2:1, 0.1% formic acid, 200 μL) using a C18 SPE cartridge and used immediately or lyophilised and stored at -20 °C.

Expression of saFabH

The *saFabH*/pET-HISTEV construct (4 μL) was transformed into an aliquot (50 μL) of BL21(DE3) (New England BioLabs) cells and set on ice for 25 minutes. The cells were then heat shocked at 42 °C for 40 seconds and set back on ice for a further 2 minutes. SOC media (100 μL) was added and the mixture was agitated at 37 °C for 1 hour. The mixture was spread on LB agar (30 μg.mL^{-1} kanamycin) and incubated overnight at 37 °C. A single transformant was used to inoculate two seed cultures of sterile LB broth (2 x 250 mL, 30 μg.mL^{-1} kanamycin) and agitated overnight at 37 °C. One of the overnight seed cultures was used to sub-culture sterile LB broth (5 x 500 mL, 30 μg.mL^{-1} kanamycin) to an OD_{600} of 0.1. The cultures were agitated at 37 °C until the OD_{600} reached 0.6, at which point expression was induced by addition of IPTG (final conc. 0.1 mM). Cells were harvested by centrifugation (30 minutes at 5000 rpm) after a further 3 hours at 30 °C and subsequently stored at -20 °C.

Purification of saFabH

N-terminal histidine-tagged *sa*FabH was purified at 4 °C by Ni-affinity chromatography followed by size exclusion chromatography. The BL21 (DE3) cell pellet expressing FabH was resuspended in lysis buffer (30 mL, 20 mM Tris-HCl pH 7.6, 300 mM NaCl, 5 mM imidazole) and lysed for 15 minutes with rounds of 30 second of sonication followed by 30 seconds of rest. The cell lysate was clarified by centrifugation (*18,000 g*, 30 minutes, 4 °C) and the cell-free extract was injected onto a HisTrap 5 mL (GE Healthcare) Ni^{2+}-affinity chromatography column pre-equilibrated in lysis buffer. The column was washed with lysis buffer (5 CV) before the histidine-tagged protein was eluted using a gradient (0-100%) of lysis buffer to elution buffer (20 mM Tris-HCl pH 7.6, 300 mM NaCl, 400 mM imidazole) over 20 CV.

Each elution fraction was analysed by SDS-PAGE, and the fractions containing His-tagged *sa*FabH were pooled, and the protein concentration was determined by a Bradford assay (Thermo Fisher). In order to remove the *N*-terminal histidine tag, a 5 mL aliquot of histidine-tagged *sa*FabH was combined with an aliquot of TEV protease (1:5 w/w) and dialysed against size exclusion mobile phase buffer (500 mL, 20 mM Tris-HCl pH 7.6, 300 mM NaCl, 10%

glycerol) at 4 °C for 16 h using 8 kDa MWCO dialysis tubing. The reaction mixture was then injected onto a HisTrap 5 mL (GE) Ni^{2+}-affinity chromatography column pre-equilibrated in lysis buffer (30 mL, 20 mM Tris-HCl pH 7.6, 300 mM NaCl, 5 mM imidazole). The flow-through was collected and concentrated to a volume of 2 mL using a 30 kDa MWCO spin-filter.

The concentrated non-histidine-tagged *sa*FabH sample was further purified by size exclusion chromatography (HiLoad Superdex 200 16/60, GE Healthcare) with an isocratic elution of mobile phase buffer (500 mL, 20 mM Tris-HCl pH 7.6, 100 mM NaCl, 10% glycerol) at 1 $mL.min^{-1}$ over 120 minutes. The saFabH eluted at approximately 70 minutes and the most concentrated fractions were pooled and flash cooled in 500 µL aliquots in liquid nitrogen before storage at -80 °C.

Expression of TEV protease

The TEV/GST fusion construct (4 µL) was transformed into an aliquot (50 µL) of BL21 (DE3) Rosetta Gami (Millipore) cells and set on ice for 25 minutes. The cells were then heat shocked at 42 °C for 40 seconds and set back on ice for a further 2 minutes. SOC media (100 µL) was added and the mixture was agitated at 37 °C for 1 hour. The mixture was spread on LB agar (30 $\mu g.mL^{-1}$ kanamycin, 25 $\mu g.mL^{-1}$ chloramphenicol) and incubated overnight at 37 °C. A single transformant was used to inoculate two seed cultures of sterile LB broth (2 x 250 mL, 30 $\mu g.mL^{-1}$ kanamycin, 25 $\mu g.mL^{-1}$ chloramphenicol) and agitated overnight at 37 °C. One of the overnight seed cultures was used to sub-culture sterile LB broth (5 x 500 mL, 30 $\mu g.mL^{-1}$ kanamycin, 25 $\mu g.mL^{-1}$ chloramphenicol) to an OD_{600} of 0.1. The cultures were agitated at 37 °C until the OD_{600} reached 0.6, at which point the temperature was reduced to 20°C and expression was induced by addition of IPTG (final conc. 0.4 mM). Cells were harvested after a further 16 h at 20 °C and subsequently stored at -20 °C.

Purification of TEV protease

N-terminal histidine-tagged TEV protease was purified at 4 °C by Ni-affinity chromatography followed by size exclusion chromatography. The BL21 (DE3) Rosetta Gami cell pellet expressing FabH was resuspended in lysis buffer (PBS, 300 mM NaCl, 10 mM imidazole, 1 mM PMSF, 1 mM benzamidine) and lysed for 15 minutes with rounds of 30 second of sonication followed by 30 seconds of rest. Excess cell matter was removed by centrifugation (*18,000 g*,

30 minutes, 4 °C) and the supernatant was injected onto a HisTrap 5 mL (GE) Ni^{2+}-affinity chromatography column pre-equilibrated in lysis buffer. The column was washed with lysis buffer (5 CV) before the histidine-tagged protein was eluted using a gradient (0-100%) of lysis buffer to elution buffer (PBS, 300 mM NaCl, 500 mM imidazole, 1 mM PMSF, 1 mM benzamidine) over 20 CV.

Each elution fraction was analysed by SDS-PAGE, and the fractions containing His-tagged TEV protease were pooled and dialysed against self-cleavage buffer (50 mM Tris-HCl pH 8.0, 300 mM NaCl, 1 mM PMSF) at 4 °C for 2 h using 8 kDa MWCO dialysis tubing in the absence of benzamidine to allow self-cleavage of the GST solubility-tag domain. The reaction was concentrated to 2 mL using a 30 kDa MWCO spin filter and further purified by size exclusion chromatography (HiLoad Superdex 200 16/60) with an isocratic elution of mobile phase buffer (50 mM Tris-HCl pH 8.0, 300 mM NaCl, 10% glycerol) at 1 mL.min^{-1} over 120 minutes. TEV protease eluted at approx. 80 minutes and the most concentrated fractions were pooled, and dialysed against storage buffer (50 mM Tris-HCl pH 8.0, 300 mM NaCl, 50% glycerol). TEV protease was flash cooled in 500 µL aliquots in liquid nitrogen and stored at -80 °C.

Supplementary Bibliography

1 K. Boukebbous, E. A. Laifa, A. De Mallmann and M. Taoufik, *IUCrData*, 2016, **1**, x161820.

2 A. A. Lebedev, P. Young, M. N. Isupov, O. V. Moroz, A. A. Vagin and G. N. Murshudov, *Acta Crystallogr. Sect. D Biol. Crystallogr.*, 2012, **68**, 431–440.

3 K. S. Gajiwala, S. Margosiak, J. Lu, J. Cortez, Y. Su, Z. Nie and K. Appelt, *FEBS Lett.*, 2009, **583**, 2939–2946.

4 P. Emsley, B. Lohkamp, W. G. Scott and K. Cowtan, *Acta Crystallogr. Sect. D Biol. Crystallogr.*, 2010, **66**, 486–501.

5 G. N. Murshudov, P. Skubák, A. A. Lebedev, N. S. Pannu, R. A. Steiner, R. A. Nicholls, M. D. Winn, F. Long and A. A. Vagin, *Acta Crystallogr. Sect. D Biol. Crystallogr.*, 2011, **67**, 355–367.
